# Logical Structures Underlying Quantum Computing

**DOI:** 10.3390/e21010077

**Published:** 2019-01-16

**Authors:** Federico Holik, Giuseppe Sergioli, Hector Freytes, Angel Plastino

**Affiliations:** 1 Instituto de Fisica (IFLP-CCT-CONICET), Universidad Nacional de La Plata, C.C. 727, 1900 La Plata, Argentina; 2 Dipartimento di Pedagogia, Psicologia, Filosofia, Università di Cagliari, Via Is Mirrionis 1, 09123 Cagliari, Italy

**Keywords:** quantum computing, non-kolmogorovian probability, quantum computational gates

## Abstract

In this work we advance a generalization of quantum computational logics capable of dealing with some important examples of quantum algorithms. We outline an algebraic axiomatization of these structures.

## 1. Introduction

Quantum computers are one of the main technological goals of our time [1] and many efforts are focused on their development. While some examples of quantum computers were actually built [2], they are still not strong enough to overcome their classical competitors: decoherence poses a threat to the problem of scaling up the number of qubits [1]. While difficult to implement, the results are promising, and a lot of interest revolves around the study of quantum algorithms both, from a theoretical standpoint and an empirical one as well. In this paper, we focus on the following problem: the characterization of the logical and algebraic structures underlying quantum algorithms. The characterization of these structures is of fundamental importance for understanding the peculiarities of quantum computation.

To describe quantum computation from a logical and algebraic point of view, many formalisms were developed (see for example [3,4,5,6,7,8,9]). We refer to the logics studied in these works as *quantum computational logics* (QCL). The QCL approach is based on the characterization of the action of quantum gates using probabilistic truth values (see also [6]).

In this work, we present a generalization of the QCL approach in which the truth values are extended to include arbitrary readings of the quantum register. This move allows us to represent quantum algorithms of practical interest in a natural way. We also generalize the QCL approach to a huge family of propositional systems, based on orthomodular lattices. Putting the emphasis in the propositional structure of the readouts of the quantum register allows for a better comparison with the classical case. Our generalization reveals that *quantum computing can be considered as a non-Kolmogorovian version of classical non-deterministic computing*, continuing the line of research proposed in [10]. From this perspective, the orthomodular lattice of projection operators of the Hilbert space is the essential algebraic structure. This lattice was termed *quantum logic* after the pioneer work of Birkhoff and von Neumann [11] (for a more recent approach, c.f. [12,13,14,15]). In the quantum case, the non-Kolmogorovian character of the probabilistic calculus involved, comes directly from the fact that there are complementary contexts in which measurements can be performed. We think that this is a very important point, because it opens the door to investigating the role of contextuality in QC in a more explicit and natural way. This is in harmony with recent studies that suggest that the essential resource allowing for the advantages of quantum computing is contextuality [16,17,18,19,20], a physical phenomenon strongly related to the non-distributive character of the lattice of quantum propositions [21]. See also [22], for a discussion of the fundamental role played by the projective geometry of the Hilbert space in quantum algorithms. Another advantage of our generalization is that of knowing a canonical form of finding quantum versions of classical formalisms and algorithms.

Finally, our approach shows how to conceive alternative forms of non-classical computing. Indeed, the general scheme presented in this manuscript could be used to compare QC with other alternative theories. A comparison of this kind, could help us to understand better the nature of quantum computing. Notice also that our formalism can be of guide for defining algorithms in physical frameworks that go beyond standard quantum mechanics. Indeed, the rigorous formulation of quantum field theory and quantum statistical mechanics require factor von Neumann algebras that go beyond the standard Type I case [23], reflecting the fact that these are (from a technical standpoint) non-equivalent probabilistic theories. Our approach can be of use in the study of quantum computing in the limit of systems with infinitely many degrees of freedom.

The paper is organized as follows. In Section 2 we review some general aspects of classical computing to motivate our further developments and present the essential aspects of the different forms of computing in a schematic way. We present our generalization of quantum computational logic in Section 3. Next, in Section 4 we review how concrete algorithms of interest in applications fall into our theoretical scheme. In Section 5 we provide the outline of an axiomatic framework for these logics, which contain classical and quantum computing as particular cases. We also discuss how these algebraic structures can be expressed in math-fashion. Finally, some conclusions are drawn in Section 6.

## 2. Classical Computing

A classical computing machine is based on bits. The bit is the elementary unit for measuring information. A physical bit (a system with only two relevant states) can take two values 0 and 1. In general, information will be stored and processed in classical computers by appealing to physical bits and operations on them. In this Section we start by reviewing the notions of deterministic and non-deterministic classical computation. Next, we describe the essential aspects of some particular models of quantum computers.

### 2.1. Deterministic Classical Computing

Any function $F:{\left\{0,1\right\}}^{N}⟶{\left\{0,1\right\}}^{M}$ can be expressed in terms of Boolean functions (see for example [24], Chapter 2). Define a Boolean function as a function $F:{\left\{0,1\right\}}^{N}⟶\left\{0,1\right\}$. A *Boolean circuit* can be represented as a composition of elementary Boolean functions. In general, a basis A of elementary functions is taken as a generating set for all other Boolean functions. One of the most important examples is the choice $A_{1}=\left\{\vee ,\wedge ,\neg \right\}$, where

**Definition** **1.** *$\vee :{\left\{0,1\right\}}^{2}⟶\left\{0,1\right\}$ with*

∨(0,0)=0

∨(0,1)=1

∨(1,0)=1

∨(1,1)=1

*$\wedge :{\left\{0,1\right\}}^{2}⟶\left\{0,1\right\}$ with*

∧(0,0)=0

∧(0,1)=0

∧(1,0)=0

∧(1,1)=1

*¬:{0,1}⟶{0,1} with*

¬(0)=1

¬(1)=0

Notice that the elements of $A_{1}$ have different *n*-arity. Any Boolean function $F:{\left\{0,1\right\}}^{N}⟶\left\{0,1\right\}$ can be written as a composition of the elementary functions belonging to $A_{1}$. Thus, the computation of *F* can be effected via a physical circuit made up physical representations of elementary Boolean functions (elementary classical gates). In other words, the hardware that allows us to compute the function *F* can be made up by electronic components representing the elementary functions ∨, ∧ and ¬, combined in a suitable way. The generalization of Boolean functions to functions $F:{\left\{0,1\right\}}^{N}⟶{\left\{0,1\right\}}^{M}$ (with M≥2) is straightforward (we refer the reader to [24] for details).

The problem of finding a suitable notion of equivalence between circuits is of major importance. In general, there is more than one way to represent a function *F* as a composition of elementary Boolean functions (and thus, very different hardware devices may compute exactly the same function). Given two functions $F,G:{\left\{0,1\right\}}^{N}⟶{\left\{0,1\right\}}^{M}$, how can we determine whether they are equal or not? Notice that similarly, we can ask if two circuits, implementing functions *F* and *G*, are essentially the same or not. This is equivalent to testing the proposition F=G. In the classical case, this can be solved in a trivial way by simply considering all possible inputs $x\in {\left\{0,1\right\}}^{N}$ and checking whether the outputs F(x) and G(x) are equal or not. Equivalently, if we do not know the functions, but we have the hardware representing them, we can compare them in a similar way, by running the two circuits and comparing the outputs. Notice that this procedure is equivalent to computing truth values of the elementary functions: if we know the truth tables of all Boolean generators, we will be able to compute the outputs of any Boolean function.

Please note that there is still more structure involved in this comparison between *F* and *G*. ${\left\{0,1\right\}}^{N}$ is a set, and the set of all its subsets $P({\left\{0,1\right\}}^{N})$ is a Boolean algebra (with the conjunction ∧ taken as set intersection, the disjunction ∨ as set union and the negation ¬ as the set theoretical complement). This is of major importance for understanding the extension to classical probabilistic computing and quantum computing. Let us discuss this with more detail. A rational agent whose function is to manage the readout of the register can only deal with (and communicate) truth values of the propositional structure defined by the power set $P({\left\{0,1\right\}}^{N})$. In this sense, the logic associated with a classical computer is represented by a Boolean algebra.

### 2.2. Non-Deterministic Classical Computing

The introduction of probabilistic steps in a computation proves to be useful for solving many particular problems [24]. Indeed, the exact solution of some problems displays high computational complexity, but this complexity can be lowered if we allow for a low rate of errors in the computation.

However, if the steps of the computation are produced in a probabilistic way, the output of an input $x\in {\left\{0,1\right\}}^{M}$ must be described by a probabilistic function $F_{x}:{\left\{0,1\right\}}^{N}⟶\left[0,1\right]$ satisfying

(1) $$\begin{array}{c} \forall \;\;x\in {\left\{0,1\right\}}^{M}\;\;\\ \sum_{y\in {\left\{0,1\right\}}^{N}}F_{x}\left(y\right)=1 \end{array}$$

Here arises an important observation. In the previous Section, we showed that the propositional structure associated with a rational agent dealing with the register was exactly the Boolean algebra $P({\left\{0,1\right\}}^{N})$. However, Equation (1) defines in a canonical way (for each $x\in {\left\{0,1\right\}}^{M}$) a Kolmogorovian probability distribution in $P({\left\{0,1\right\}}^{N})$ as follows:

$$\mu_{F_{x}}:P\left({\left\{0,1\right\}}^{N}\right)\to \left[0,1\right]$$

such that:

1. $\mu_{F_{x}}\left(\emptyset \right)=0$
2. $\mu_{F_{x}}\left(A^{c}\right)=1-\mu_{F_{x}}\left(A\right)$
3. For any disjoint $X,Y\in P({\left\{0,1\right\}}^{N})$ we have
  $$\mu_{F_{x}}\left(X⋃Y\right)=\mu_{F_{x}}\left(X\right)+\mu_{F_{x}}\left(Y\right)$$

Thus, the classical propositional structure of the register implies that there will be a classical probabilistic distribution for the propositions communicated by a rational agent dealing with the readouts.

### 2.3. Quantum Computing in a Schematic Way

Before we continue, it is useful to give some definitions. States of a quantum system can be represented by so-called *density operators*, which are positive and trace class self-adjoint operators of trace one, acting on a separable Hilbert space H. We call C(H) to the set of all density operators. A density operator ρ is said to be *pure* if $\rho^{2}=\rho$ and *mixed* otherwise. An operator *U* acting on a separable Hilbert space is said to be *unitary* iff $UU^{†}=U^{†}U=1$ (here, $U^{†}$ represents the adjoint of the operator *U*). Quantum gates will be represented by unitary operators. As is well known, if *U* is unitary, the map $E\left(\rho \right)=U\rho U^{†}$ maps density operators into density operators.

Let us try to summarize how a quantum computer works. Suppose that our quantum hardware has *N* qubits. In this case, the Hilbert space is $H={⨂}^{N}C^{2}$. A basic general description of all the central operations needed to perform a quantum algorithm can be given as follows.

Step 1. Chose an initial state represented by a density operator ρ for the qubits (notice that ρ can be taken to be mixed [25]). This state could be just the density operator associated with the vector |0〉⊗|0〉⊗⋯⊗|0〉 or any other desired state. In most situations of practical interest, the computational basis plays a key role in defining the inputs and the outputs of the algorithm. Let us denote the computational basis by $B_{0}$.
Step 2. Apply a collection of gates represented by unitary operators ${\left\{U_{i}\right\}}_{i=1,\ldots ,n}$ to reach a desired final state $\sigma =U_{n}\cdots U_{2}U_{1}\rho U_{1}^{†}U_{2}^{†}\cdots U_{n}^{†}$.
Step 3. Perform a measurement on the system when state σ is reached, check the result obtained, and depending on the result, stop the process, or continue following the pertinent protocol if necessary (which will involve a similar, or, in some cases, the same- process). Notice that the measurement process involves the choice of a measurement basis. This process has associated a probability (dictated by Born’s rule). The probability of success for an algorithm is related to the probability of occurrence of a certain outcome (or collection of them). This outcome should be of interest for the successful computation involved in the protocol that one is following. Notice that a subspace containing the desired results always exists. The pertinent probability of success is related to the probability for the event of interest to happen.

It is illustrative to compare now the possible readouts of a rational agent *R* dealing with a quantum computer. Like in the classical probabilistic case, the possible observations are not restricted to the computational basis $B_{0}$. Notice that $B_{0}$ defines a Boolean algebra of operators in a canonical way as follows. First, consider the set $P_{0}$ of all possible orthogonal projection operators corresponding to the elements of $B_{0}$. The commutant of a set *C* of bounded operators acting on a Hilbert space is defined as $C^{\prime }:=\left\{x\in B\left(H\right)\;\;|\;\;\left[x,y\right]=0\;\;\forall \;\;y\in C\right\}$. The double commutant of *C* is defined as ${\left(C^{\prime }\right)}^{\prime }$. Now, take the double commutant of $P_{0}$ to define the set $P_{B_{0}}:={\left(P_{0}\right)}^{\prime \prime }$. It is easy to check that $P_{B_{0}}:={\left(P_{0}\right)}^{\prime \prime }$ is a Boolean algebra. The projection operators in $P_{B_{0}}$ form the propositional structure associated with the computational basis. However, as in the classical case, the measurements of the rational agent will not be generally restricted, to this set of propositions. Indeed, by applying rotations on the output state, *R* can measure other properties associated with the final quantum state. In case that, due to restrictions in the hardware, the readouts are only performed on the computational basis, a measurement in a different basis can be implemented by a rotation *U* applied on the quantum state (using the equivalence $\mathrm{Tr}\left(\rho U^{†}PU\right)=\mathrm{Tr}\left(U\rho U^{†}P\right)$). As it is well known, the set of possible propositions to be tested is formed by the set P(H) of all orthogonal projection operators acting in H. The collection P(H) is a modular non-distributive lattice for finite dimensional Hilbert spaces and an orthomodular one for infinite dimensional ones. The final quantum state—represented by the density operator σ—defines a quantum probability distribution

$$P_{\sigma }:P\left(H\right)⟶\left[0,1\right]$$

given by $P_{\sigma }\left(P\right)=\mathrm{Tr}\left(\sigma P\right)$ for all P∈P(H). It possesses the following properties:

1 $P_{\sigma }\left(0\right)=0$ (0 is the null operator).
2 $P_{\sigma }\left(P^{⊥}\right)=1-P_{\sigma }\left(P\right)$, for all P∈P(H).
3 For any family ${\left(P_{j}\right)}_{j\in J}\subset P\left(H\right)$ consisting of orthogonal projections for which $P_{j_{1}}P_{j_{2}}=0$ when $j_{1}\neq j_{2}$, the following equality holds:
  $$P_{\sigma }\left(⋁P_{j}\right)=\sum_{j\in J}P_{\sigma }\left(P_{j}\right).$$

Observe that, since the Hilbert space H is supposed to be separable, the family may be taken to be finite or countable. Notice also that the equality P∨Q+P∧Q=P+Q is true provided PQ=QP=P∧Q. Here the operations P∨Q and P∧Q are appropriately defined within the $W^{*}$-algebra (or von Neumann algebra) under discussion. In case that this $W^{*}$-algebra coincides with B(H), i.e., the algebra of all bounded linear operators on the Hilbert space H, then the operator P∧Q is the orthogonal projection on the subspace PH∩QH, and P∨Q is the orthogonal projection on the closure of the subspace PH+QH. Of course, similar equalities hold for families of commuting orthogonal projections. We always have $P^{⊥}=1-P$.

While the properties of a quantum probability distribution may resemble the properties of a Kolmogorovian one, there is a radical difference. The probability distribution associated with a classical probabilistic algorithm is defined in a Boolean algebra, but the probability distribution associated with a quantum one is defined in an orthomodular lattice [26]. The differences between these probability theories has been extensively studied in the literature (see for example [12,23,27]). For more discussion on this subject see [28,29,30], where the authors propose applications of non-Kolmogorovian probability theory outside of the quantum domain.

## 3. Logics Associated with Quantum Algorithms

In this Section we introduce our proposal for generalizing quantum computational logics. We start with the characterization of compositional gates and end up with a generalization of probabilistic truth values.

### 3.1. The Algebraic Structure of Quantum Logical Gates

In a real quantum computer, a general quantum gate is constructed by adequately composing elementary ones. In theoretical quantum computing, certain sets of universal gates are used. As examples, we can take the quantum Toffoli and the Hadamard gates [31]; another example is given by CNOT, Hadamard, and controlled phase gates. The important point to remark is the fact that we use, as a starting point, a set of generating gates $G=\{G_{1},\ldots ,G_{N}\}$ (with finite elements). In the above examples we have: $G_{1}=\left\{T,H\right\}$ and $G_{2}=\left\{CNOT,H,R_{\phi }\right\}$. The native gates of the hardware presented in [2] are given by $G_{3}=\left\{XX,R_{\phi }\right\}$. In this paper, we concentrate on $G_{1}$, but a similar analysis could be carried out for $G_{2}$ and $G_{3}$.

Given a generating set of gates *G*, all physically implementable gates will be given by successive applications of the elements of *G*. This means that any actual gate implemented in the quantum computer will have the form: $U=U_{n}\ldots U_{1}$, where $U_{i}\in G$. Let us call to these compositions of gates (in analogy with the classical case) *quantum polynomial gates*. Denote by P(G) the set of all possible quantum polynomial gates. If *G* is formed by a universal set of gates, then P(G) will be dense in $U_{N}\left(H\right)$ (the set of unitary operators acting on H). From a theoretical perspective, this is all we need to implement any desired quantum algorithm.

Any component of a quantum algorithm will be described as a succession of polynomials in P(G), and eventually, a reading (measurement) described by a projection onto a subspace or a family of them, with their associated probabilities computed using the Born rule. Notice that P(G) and its topological closure are the quantum versions of the logic of a classical computer. In the classical version, we have a functional calculus based on Boolean functions. The Boolean character is related to the fact that these functions commute. In the quantum version, we have a non-commutative matrix calculus instead.

An important problem in the logical approach to classical computation is given by testing functional equations. This problem can be solved by appealing to truth tables of elementary Boolean functions, such as ∨, ∧ and ¬, as explained in Section 2. However, in the quantum version, the existence of non-compatible context and indeterminate processes leads us to a different notion of truth, based on probabilistic and contextual truth values. In the following Subsection, we explain how this works.

### 3.2. Probabilistic Truth Values

We start by defining equivalences between gates as follows:

**Definition** **2.** *Given two quantum gates U and V, we say that*

- *U is equivalent to V with respect to ρ∈C(H) and P∈P(H) (and we denote it by $U\equiv_{P}^{\rho }V$) if and only if*
  $$Tr\left(U\rho U^{†}P\right)=Tr\left(V\rho V^{†}P\right)$$
  *For a given quantum gate U, we call* probabilistic truth value associated with *U* with respect to P∈P(H)*the real number $Tr(U\rho U^{†}P)$.*
- *U is equivalent to V with respect to ρ∈C(H) (and we denote it by $U\equiv^{\rho }V$) if and only if*
  $$Tr\left(U\rho U^{†}P\right)=Tr\left(V\rho V^{†}P\right)$$
  *for all P∈P(H).*
- *U is equivalent to V with respect to P∈P(H) (and we denote it by $U\equiv_{P}V$) if and only if*
  $$Tr\left(U\rho U^{†}P\right)=Tr\left(V\rho V^{†}P\right)$$
  *for all ρ∈C(H).*
- *U is equivalent to V (and we denote it by U≡V) if and only if*
  $$Tr\left(U\rho U^{†}P\right)=Tr\left(V\rho V^{†}P\right)$$
  *for all ρ∈C(H) and P∈P(H).*

Notice that neither $U\equiv^{\rho }V$ nor $U\equiv_{P}^{\rho }V$ imply that U=V. On the contrary, U≡V implies U=V. We have included this last (trivial) definition just to easily illustrate the fact that $U\equiv^{\rho }V$ and $U\equiv_{P}^{\rho }V$ are relaxations of the identity relationship. Indeed, we can summarize this as follows:

$$U=V⟺U\equiv V⟹U\equiv^{\rho }V⟹U\equiv_{P}^{\rho }V.$$

It is easy to find counterexamples showing that the converse implications are not valid.

The above definitions of equivalence between logical gates have associated the following probabilistic truth values:

**Definition** **3.** *Given two gates U and V, we say that*

- *For a given quantum gate U, we call* probabilistic truth value associated with *U* in the context *P* and state ρ *the real number $Tr(U\rho U^{†}P)$.*
- *For a given quantum gate U, we call* probabilistic truth values associated with *U* in the context *P* the family of real numbers $Tr(U\rho U^{†}P)$, where ρ∈C.
- *For a given quantum gate U, we call* probabilistic truth values associated with *U* in the state ρ the family of real numbers $Tr(U\rho U^{†}P)$, where P∈P(H).

Notice that the above definitions have associated a notion of implication between gates:

**Definition** **4.** *Given two gates U and V, we say that*

- *U≤V with respect to ρ∈C(H) and P∈P(H) (and we denote it by $U\leq_{P}^{\rho }V$) if and only if*
  $$Tr\left(U\rho U^{†}P\right)\leq Tr\left(V\rho V^{†}P\right)$$
- *U≤V with respect to ρ (and we denote it by $U\leq^{\rho }V$) if and only if*
  $$Tr\left(U\rho U^{†}P\right)\leq Tr\left(V\rho V^{†}P\right)$$
  *for all P∈P(H).*
- *U≤V with respect to P (and we denote it by $U\leq_{P}V$) if and only if*
  $$Tr\left(U\rho U^{†}P\right)\leq Tr\left(V\rho V^{†}P\right)$$
  *for all ρ∈C(H).*

It is also important to notice that the “$\equiv_{P}^{\rho }$" relationship is coincident with the notion of probabilistic truth values of previous publications. For example, in [5,7], the probabilistic truth value of the Toffoli gate is defined as:

(2) $$p\left(\rho ⊗\sigma \right)=Tr(T\rho ⊗\sigma ⊗|0\rangle \langle 0|T)\left(I⊗I⊗P_{1}\right))$$

where $P_{1}:=\left|1\rangle \langle 1\right|$.

Another important issue is at stake here. The notion of probabilistic truth value outlined in Definition 5 contains the Boolean truth notion as a particular case as follows. We use as starting state the elements of the computational basis. Then, implement matrix versions of the usual classical gates (as for example, Toffoli). Use as a projection operator the projection associated with any element of the computational basis (or more generally, one may use the Boolean lattice $P_{B_{0}}$, defined in Section 2.3). This procedure yields a Boolean calculus which is isomorphic to the usual one in reversible classical computation.

With the above definitions, we can define the truth value associated to each measurement outcome (i.e., the reading process) of any quantum protocol. The final truth value of the protocol, associated to the probability of occurrence of the success subspace, will be related to the probability of success of the algorithm.

As in the classical case, we need to test the equivalence between different sets of gates. This can be done in a natural way by appealing to the $\equiv^{\rho }$ and $\equiv_{P}^{\rho }$ relations. Indeed, if our aim is to compare the action of two sets of gates regarding a definite subspace of success, we must use the $\equiv_{P}^{\rho }$ relationship. If our aim is to compare two gates regarding the unitary process before any reading (measurement), we must use the stronger relation $\equiv^{\rho }$.

Notice that this last definition of equivalence (the one given by $\equiv^{\rho }$) is the generalization of the truth table to the Boolean setting. Let us elaborate a little bit on this notion. In a classical setting, if we aim to compare between logical circuits (defined as compositions of Boolean functions), all we must do is to list truth tables on each side of the equation. A similar remark follows for non-deterministic classical computation: we must test the equivalence by evaluating the probability of each output on each side of the equation. In both cases (deterministic and no-deterministic), if these numbers are coincident, we speak about logically equivalent algorithms.

However, now, as remarked above, we have infinitely many contexts in the quantum case (indeed, the whole P(H) is available). This forces us to test the equivalence (equality in a logical circuit equation) in several different contexts. Let us illustrate this statement with a concrete example. Suppose that we have two gates *U* and *V*, and that we want to check their equivalence with respect to a reference state ρ. Thus, we must compare $Tr\left(U\rho U^{†}P\right)=Tr\left(V\rho V^{†}P\right)$ for a suitably chosen set of projection operators (in principle, there are infinitely many of them, but in practice, only a reduced family of them is needed, depending on the dimensionality of the Hilbert space. This is a well-studied question). Notice that $Tr\left(U\rho U^{†}P\right)=Tr\left(V\rho V^{†}P\right)$ is equivalent to $Tr\left(\rho U^{†}PU\right)=Tr\left(\rho V^{†}PV\right)$. To test this equation (and to give the problem a form similar to the classical one) we can choose a suitable family of orthonormal bases, each defining a different measurement context. Each one of these bases defines its own “computational basis”. Thus, instead of checking the probabilities in one single Boolean context (a set of outputs) as in the non-deterministic classical case, what we are doing here is to perform the same procedure for all possible quantal contexts defined by the chosen set of bases (or at least, for all possible contexts of interest for the problem we want to solve). All these properties reveal that quantum computing is the non-commutative version of classical non-deterministic computation (as expected!), and that the relationship defined by $\equiv^{\rho }$ is nothing but the generalization of the truth tables to the non-commutative setting.

The above definitions introduce different equivalence notions of gates. In addition, we can compute a kind of quotient of P(G) with regards to this equivalence relationships (i.e., we can compute the quotient spaces $P\left(G\right)/\equiv_{P}^{\rho }$ and $P\left(G\right)/\equiv^{\rho }$). Notice that P(G)/≡ is meaningless, because P(G)/≡ equals P(G). Each class of $P\left(G\right)/\equiv_{P}^{\rho }$ and $P\left(G\right)/\equiv^{\rho }$ contains equivalent gates for the different purposes defined by the different equivalence relations.

## 4. Examples of Quantum Algorithms

Now we show how our approach can accommodate some of the most important quantum algorithms.

### 4.1. Deutsch-Jozsa Algorithm

Let us examine first the Deutsch-Jozsa algorithm [1]. In this case, the task is to determine if a function *f* is constant or balanced. There are four functions from {0,1}⟶{0,1}, namely:

(3) $$\begin{array}{c} f_{1}\left(0\right)=0\;\;\;f_{1}\left(1\right)=1\\ f_{2}\left(0\right)=1\;\;\;f_{2}\left(1\right)=0\\ f_{3}\left(0\right)=0\;\;\;f_{3}\left(1\right)=0\\ f_{4}\left(0\right)=1\;\;\;f_{4}\left(1\right)=1 \end{array}$$

Thus, we have two classes: $C=\{f_{1},f_{2}\}$ and $B=\{f_{3},f_{4}\}$, and we must determine if the unknown function *f* belongs to *B* or to *C*. Let us see now how this can be reduced to the steps outlined in Section 2.3.

Step 1. In the first step, prepare the quantum state |0〉|1〉.
Step 2. Next, the Hadamard operator is applied to both qubits yielding the state:
  $$\frac{1}{2}(|0\rangle +|1\rangle )(|0\rangle -|1\rangle ).$$
  The quantum implementation of the function *f* (which establishes the connection between the classical problem and the quantum computation) will be given by a quantum operator such that it maps |x〉|y〉 to |x〉|f(x)⊕y〉. Applying this function to the state gives
  $${\left(-1\right)}^{f(0)}\frac{1}{2}(|0\rangle +{\left(-1\right)}^{f(0)⊕f(1)}|1\rangle )(|0\rangle -|1\rangle ).$$
  Now, applying the Hadamard transformation again to the first qubit we get:
  $$|\psi \rangle ={\left(-1\right)}^{f(0)}\frac{1}{2}(\left(1+{\left(-1\right)}^{f(0)⊕f(1)}\right)|0\rangle +\left(1-{\left(-1\right)}^{f(0)⊕f(1)}\right)|1\rangle )(|0\rangle -|1\rangle ).$$
Step 3. The next step consists in determining the projection of the above state to the subspaces represented by projection operators |0〉〈0|⊗1 and |1〉〈1|⊗1.

### 4.2. Determination of a Function’s Period

The determination of the period of a periodic function *f* lies at the heart of the Shor and Simon quantum computation algorithms [1]. The objective now is to determine the period of a function $f:Z_{N}⟶Z$, such that f(x+r)=f(x) for all *x*. It is assumed that the function does not take the same value twice in the same period.

Step 1. Start the computer by generating -using the usual procedure- the state:
  (4) $$|f\rangle =\frac{1}{\sqrt{N}}\sum_{x=0}^{N-1}|x\rangle |f\left(x\right)\rangle .$$
  It is not possible to extract the period yet. Even if we measure the value of the second register and obtain the value $y_{0}$, we will end up with the following state in the first register (with $x_{0}$ the smallest *x* such that $f\left(x\right)=y_{0}$ and N=Kr):
  (5) $$|\psi \rangle =\frac{1}{\sqrt{K}}\sum_{k=0}^{K-1}|x_{0}+kr\rangle .$$
  However, |ψ〉 does not give us information about *r* yet.
Step 2. To obtain the period, it is necessary to apply the quantum Fourier transform (QFT), which is a unitary matrix with entries
  (6) $$F_{ab}=\frac{1}{\sqrt{N}}\exp^{2\pi i\frac{ab}{N}}.$$
  By applying the QFT to |ψ〉 we obtain
  (7) $$F|\psi \rangle =\frac{1}{\sqrt{r}}\sum_{j=0}^{r-1}\exp^{2\pi i\frac{x_{0}j}{r}}|j\frac{N}{r}\rangle .$$
Step 3. Finally, a measurement is performed in the basis $\left\{|j\frac{N}{r}\rangle \right\}$, and using the result it is possible to determine the period of the function as follows. The obtained value *c* will be such that $c=j\frac{N}{r}$, for some 0≤j≤r−1. Then, $\frac{c}{j}=\frac{N}{r}$, and if *j* is coprime with *r*, it will be possible to determine *r*. The success of the algorithm depends on the fact that *j* and *r* will be coprimes with a large enough probability.

## 5. Axiomatization of the Quantum Computational Logic

In the classical case, we know that the functions are given by a commutative calculus generated by the elementary Boolean functions ∨, ∧ and ¬. Thus, the axiomatization of a classical computational logic can be given in terms of the axioms defining a Boolean algebra. Is it possible to proceed in an analogous way for our notion of quantum computational logics? We outline an answer to this question below.

Notice first that the classical connectives ∨, ∧ and ¬ have the natural interpretation given by:

- ∨ is the operation of disjunction in our natural language.
- ∧ is the operation of conjunction in our natural language.
- ¬ is the operation of negation in our natural language.

However, quantum computers operate in a very different way. If we consider as elementary gates the set $G_{3}$, we have the following semantical/physical interpretation:

- $H_{i}$: generates a superposition in qubit *i*.
- CNOT: flips the value of the second qubit if the control qubit is 1; do nothing otherwise.
- $R_{\phi }$ adds a phase of ϕ in one of the terms of the superposition.

Notice the following: all truth values of a classical probabilistic calculus are computed regarding classical propositions. However, these propositions are nothing but the elements of a Boolean algebra. As an example, consider a computer with *N* bits. Thus, all possible inputs are given by elements of the set ${\left\{0,1\right\}}^{N}$ (and also the outputs). Thus, all possible readings (measurements) in the output of a computation are given by elements of ${\left\{0,1\right\}}^{N}$, and all possible propositions are nothing but conjunctions, disjunctions, and negations of this outcome set. In other words, all elementary propositions are given by the elements of the Boolean algebra $P({\left\{0,1\right\}}^{N})$. The nature of the classical (deterministic) computing process assigns a valuation to the set {0,1} to each element of the set $P({\left\{0,1\right\}}^{N})$. Non-deterministic classical computation instead, assigns probability values in the interval [0,1].

The quantum setting, despite of its formidable appearance, is completely analogous. We have stressed above that the quantum calculus contains the classical one as a special case. This is totally reasonable: quantum mechanics defines a probabilistic classical theory for each context. In other words, a quantum state can be seen as a collection of classical probability distributions pasted in a harmonic way using the density operator associated with that state [12]. What is thus the analogous of a reading of a quantum register? The answer was given by von Neumann in the first axiomatization of the formalism: each elementary reading (i.e., any possible empirically testable proposition) is represented by a projection operator P∈P(H). Thus, we have a very clear operational interpretation of the equivalence relations and truth values defined in Definition 5: what we are doing here is to compute the truth value of a quantum state regarding a particular proposition. This notion of truth in quantum computation is all that we need to compare different sets of gates and the success probability of each quantum algorithm.

Another important thing to remark is that the calculus defined by the matrices involved in a quantum case is not commutative. Thus, we can anticipate that the axiomatization will not involve a Boolean algebra.

Thus, to define a suitable algebraic axiomatics for quantum computation, all we must do is to consider: (i) a set L of empirically testable propositions (which are intended to represent all possible readings of the quantum register). It is natural to demand that L should be an orthomodular lattice (as we show below, this covers the classical and quantum cases as well); (ii) a set of states C assigning definite probabilities to each element of L (these can be defined in the usual way as σ-additive probabilities) [12]; (iii) an elementary set *G* of operations acting by automorphisms in L. Remember that an automorphism of a logic is defined as a bijective map U:L⟶L satisfying (a) U(0)=0 and U(1)=1, (b) for any denumerable sequence $X_{1},X_{2},\ldots$ we have $U\left({⋁}_{n}X_{n}\right)={⋁}_{n}U\left(X_{n}\right)$ and $U\left({⋀}_{n}X_{n}\right)={⋀}_{n}U\left(X_{n}\right)$ and (c) for all X∈L, $U\left(X^{⊥}\right)={\left(U\left(X\right)\right)}^{⊥}$. The set *G* generates the collection P(G) of logical polynomials (by composition). The set Ξ=〈L;C;G〉 will be called a *generalized computational scheme*. In the rest of this section, we make extensive use of the following notation: given an automorphism *U* acting on L, define the action U(ν) of *U* on the state ν as U(ν)(X):=ν(U(X)), for all X∈L. Using Ξ we can define P(G) (that determines the set of all possible logical gates) and the notions of probabilistic truth value relative to a context and equivalence of logical gates as follows:

**Definition** **5.** *Given two gates U,V∈P(G), we say that*

- *U is equivalent to V with respect to ν∈C and X∈L (and we denote it by $U\equiv_{X}^{\nu }V$) if and only if*
  $$\mu \left(X\right)=\mu^{\prime }\left(X\right)$$
  *where μ(−)=U(ν)(−) and $\mu^{\prime }\left(-\right)=V\left(\nu \right)\left(-\right)$.*
- *U is equivalent to V with respect to ν (and we denote it by $U\equiv^{\nu }V$) if and only if*
  $$\mu \left(X\right)=\mu^{\prime }\left(X\right)$$
  *for all X∈L.*
- *U is equivalent to V (and we denote it by U≡V) if and only if $\mu \left(X\right)=\mu^{\prime }\left(X\right)$ for all ν∈C and X∈L.*

We can also define the notion of *generalized protocol* using the following steps:

Step 1. Chose an initial reference state ν∈C (this state is intended to be the same for all possible algorithms, and is interpreted as the initial state of the devise).
Step 2. Apply a collection of gates ${\left\{U_{i}\right\}}_{i=1,\ldots ,n}$ to reach a desired final state $\mu \left(-\right)=\left(U_{n}\cdots U_{2}U_{1}\right)\left(\nu \right)\left(-\right)$, possessing the properties needed to perform the desired computation (answer the question that we need to answer).
Step 3. Perform a measurement on the system when the state μ is reached, check the result obtained, and depending on the result, stop the process, or continue the protocol if necessary (which will involve a similar -in some cases, the same- process).

The intended interpretations of the above notions are similar to those of classical and quantum algorithms. We recover classical computation by setting L=B (where B is a Boolean algebra) and quantum computation when L=P(H) (using the concomitant definitions of initial reference state, probabilities, etc.).

As in the quantum case, we can give definitions of probabilistic truth values:

**Definition** **6.** *Given two gates U and V, we say that*

- *For a given generalized gate U, we call* probabilistic truth value associated with *U* in the event X∈L and initial state ν*the real number U(ν)(X).*
- *For a given generalized gate U, we call* probabilistic truth values associated with *U* in the event X∈L*the family of real numbers U(ν)(X), where ν∈C.*
- *For a given generalized gate U, we call* probabilistic truth values associated with *U* in the state ν *the family of real numbers U(ν)(X), where X∈L.*

Notice that the above definitions have associated a notion of implication between gates:

**Definition** **7.** *Given two gates U and V, we say that*

- *U≤V with respect to ν∈C and X∈L (and we denote it by $U\leq_{X}^{\nu }V$) if and only if*
  U(ν)(X)≤V(ν)(X)
- *U≤V with respect to ν (and we denote it by $U\leq^{\nu }V$) if and only if*
  U(ν)(X)≤V(ν)(X)
  *for all X∈L.*
- *U≤V with respect to X∈L (and w denote it by $U\leq_{X}V$) if and only if*
  U(ν)(X)≤V(ν)(X)
  *for all ν∈C.*

The effect of the Hadamard gate in the computational basis is to generate a superposition out of the input qubit. Thus, it is relevant for our construction to describe how superpositions are defined in arbitrary orthomodular lattices (we follow [32], Chapter III, Section 4). Given an orthomodular lattice L, let D be a collection of states in L. Then, a state ν is a *superposition* of the states in D, if and only if, for all X∈L we have

(8) ∀μ∈D(μ(X)=0)⟹ν(X)=0

The above definition coincides with the usual one for the case of the lattice of projection operators acting on a Hilbert space and pure states. In Boolean algebras, no pure state can be a superposition of other pure states. As automorphisms of a logic are angle-preserving (i.e., they are straightforward generalizations of the rotations acting on the projective geometry associated with a Hilbert space), their effect on a given set of propositions will be, in general, to generate superpositions. This fact guarantees that we will be able to recover a quantum-like computation rich enough so as to display contextuality and entanglement (as is the case for example, with factor von Neumann algebras, for which a version of the Kochen-Speker theorem can be proved [21]; for the study of correlations in the algebraic approach see [33]).

## 6. Conclusions

In this work we have presented a generalization of quantum computational logics capable of dealing with some important examples of quantum algorithms. We show that our constructions generalize previous studies on the subject. In Section 5 we have outlined an axiomatization for quantum computational logics. Our developments lead to new problems related to the algebraic characterization of computational gates in a non-commutative setting. They also open the door for further generalization of the notion of *algorithm*, beyond the classical and standard quantum mechanical formalisms. 
